# Interleukin-17 Retinotoxicity Is Prevented by Gene Transfer of a Soluble Interleukin-17 Receptor Acting as a Cytokine Blocker: Implications for Age-Related Macular Degeneration

**DOI:** 10.1371/journal.pone.0095900

**Published:** 2014-04-29

**Authors:** Daniel Ardeljan, Yujuan Wang, Stanley Park, Defen Shen, Xi Kathy Chu, Cheng-Rong Yu, Mones Abu-Asab, Jingsheng Tuo, Charles G. Eberhart, Timothy W. Olsen, Robert F. Mullins, Gary White, Sam Wadsworth, Abraham Scaria, Chi-Chao Chan

**Affiliations:** 1 Immunopathology Section, Laboratory of Immunology, National Eye Institute, National Institutes of Health, Bethesda, Maryland, United States of America; 2 School of Medicine, Johns Hopkins University, Baltimore, Maryland, United States of America; 3 Zhongshan Ophthalmic Center, Sun Yat-sen University, Guangzhou, China; 4 Howard Hughes Medical Institute, Chevy Chase, Maryland, United States of America; 5 Molecular Immunology Section, Laboratory of Immunology, National Eye Institute, National Institutes of Health, Bethesda, Maryland, United States of America; 6 Histology Core, Laboratory of Immunology, National Eye Institute, National Institutes of Health, Bethesda, Maryland, United States of America; 7 Department of Pathology, Johns Hopkins University, Baltimore, Maryland, United States of America; 8 Department of Ophthalmology, Emory University, Atlanta, Georgia, United States of America; 9 Department of Ophthalmology and Visual Sciences, University of Iowa Carver College of Medicine, Iowa City, Iowa, United States of America; 10 Genzyme Corporation, Framingham, Massachusetts, United States of America; Oregon Health & Science University, United States of America

## Abstract

Age-related macular degeneration (AMD) is a common yet complex retinal degeneration that causes irreversible central blindness in the elderly. Pathology is widely believed to follow loss of retinal pigment epithelium (RPE) and photoreceptor degeneration. Here we report aberrant expression of interleukin-17A (IL17A) and the receptor IL17RC in the macula of AMD patients. *In vitro*, IL17A induces RPE cell death characterized by the accumulation of cytoplasmic lipids and autophagosomes with subsequent activation of pro-apoptotic Caspase-3 and Caspase-9. This pathology is reduced by siRNA knockdown of *IL17RC*. IL17-dependent retinal degeneration in a mouse model of focal retinal degeneration can be prevented by gene therapy with adeno-associated virus vector encoding soluble IL17 receptor. This intervention rescues RPE and photoreceptors in a MAPK-dependent process. The IL17 pathway plays a key role in RPE and photoreceptor degeneration and could hold therapeutic potential in AMD.

## Introduction

Age-related macular degeneration (AMD) is the leading cause of central irreversible blindness in people age 55 and older [1], [2]. The disease is characterized earliest by the appearance of extracellular material (drusen) in macular Bruch’s membrane and external to the retinal pigment epithelium (RPE). Many patients develop one of two phenotypes: geographic atrophy (GA or “dry AMD”) characterized by atrophy of RPE and photoreceptors, or neovascular AMD (nAMD or “wet AMD”) characterized by choroidal neovascularization (CNV). AMD risk factors include aging, smoking, and genetics, particularly a common polymorphism in complement factor H. RPE damage is an inciting event believed to be the primary pathological insult leading to photoreceptor atrophy [3]. The specific mechanism is largely unknown.

Growing clinical suspicion is emerging for involvement of interleukin-17A (IL17A) in AMD. Complement component C5a, found in AMD patient serum and in drusen [4]–[6], induces IL17A secretion from CD4^+^ T cells [7]. IL17A in the serum and hypomethylation of the *IL17RC* promoter in twins with discordant AMD status has been shown [7], [8]. Subsequently, we noted that the anti-inflammatory agent TSG-6 halted *Ccl2^−/−^*/*Cx3cr1^−/−^*/*Crb1^rd8^* (DKO/*rd8*) retinal degeneration and found that treated retinas expressed less *Il17a* [9]. IL17A has been shown to drive autoimmune diseases including rheumatoid arthritis and experimental autoimmune uveitis [10].

Since AMD patients present without systemic inflammation, we hypothesized that local IL17 signaling was retinotoxic in AMD. We first characterized the expression of IL17A and IL17RC in the AMD retina and then determined the cytokine’s effect on RPE viability *in vitro*. Finally, we knocked down IL17 signaling in a mouse model of focal retinal degeneration (DKO/*rd8)* [11], [12] using an adeno-associated viral vector encoding a soluble IL17 receptor.

## Results

### Aberrant Levels of IL17A and IL17RC in the AMD Macula

We isolated RNA from microdissected macular lesions of 107 paraffin-embedded eyes from 67 advanced stage AMD donors (35 GA, 72 nAMD) and 17 eyes from 10 age-matched control donors collected over the past 50 years from Johns Hopkins and NEI. Only 41 (38.3%) of AMD eyes (9 GA, 32 nAMD) and 8 (47.1%) of control eyes yielded quantifiable results. We also detected transcripts in sub-macular choroid button of 9 control and 17 AMD donor eyes.

**Figure 1:**
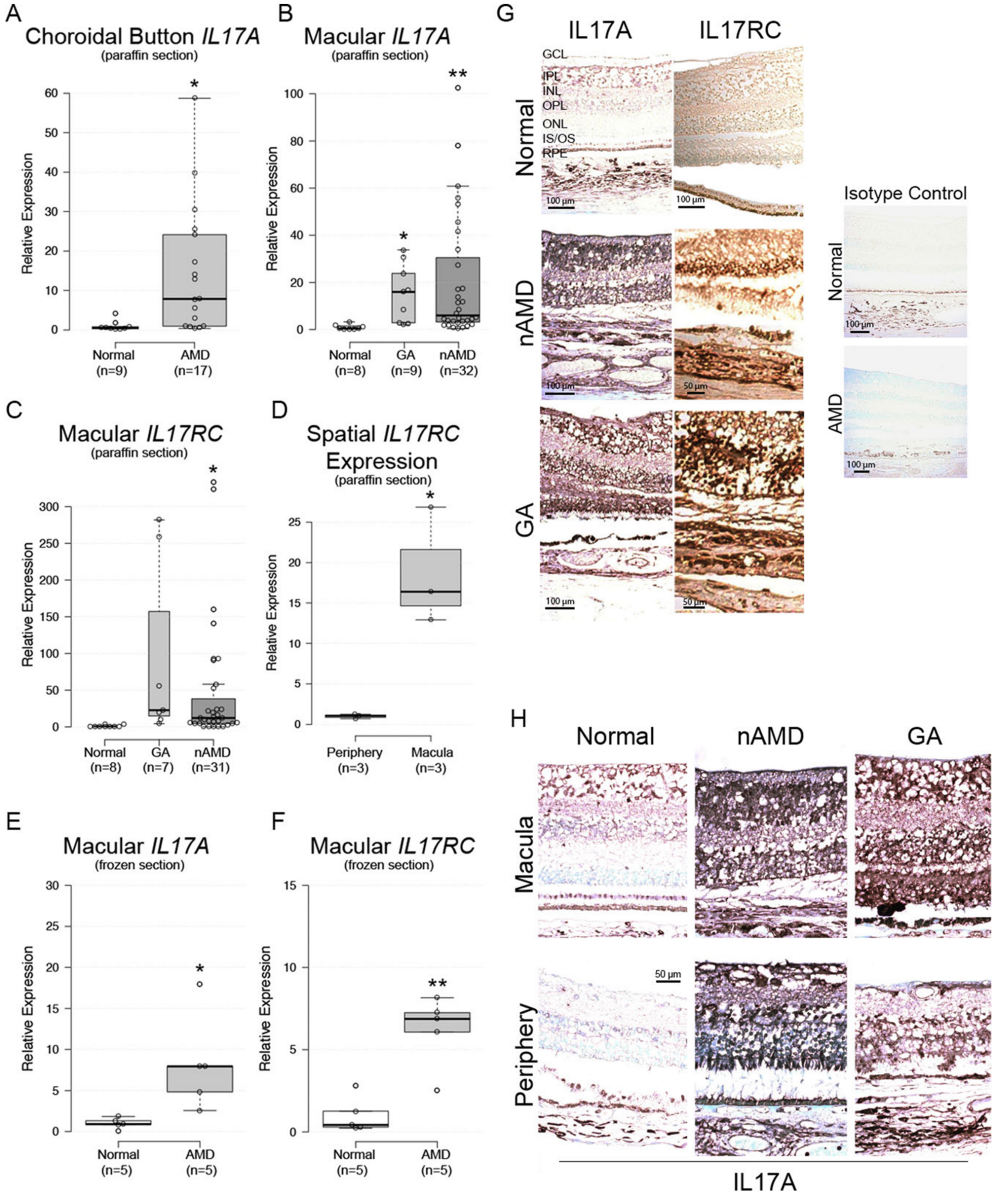
*IL17A* and *IL17RC* expression in AMD tissue. *IL17A* mRNA expression in paraffin-embedded (A) sub-macular choroid button and (B) macula. (C) *IL17RC* mRNA expression in paraffin-embedded macula. (D) *IL17RC* mRNA expression in macula vs. periphery of 3 donors. (E, F) Macular *IL17A* and *IL17RC* mRNA were verified in paraformaldehyde-fixed fresh frozen tissue. (G) Immunohistochemical detection of IL17A and IL17RC in paraffin-embedded macular sections. Isotype controls lacked primary antibodies. (H) Comparison of macular and peripheral IL17A immunostains. For box plots: center lines show the medians; box limits indicate the 25th and 75th percentiles as determined by R software; whiskers extend 1.5 times the interquartile range from the 25th and 75th percentiles, outliers are represented by dots; width of the boxes is proportional to the square root of the sample size; data points are plotted as open circles; sample numbers are indicated beneath each column. GA = geographic atrophy; nAMD = neovascular AMD; GCL = ganglion cell layer; IPL = inner plexiform layer; INL = inner nuclear layer; OPL = outer plexiform layer; ONL = outer nuclear layer; IS/OS = inner/outer segment; RPE = retinal pigment epithelium. *: P<0.05; **: P<0.005; ***:P<0.0001.

Macular *IL17A* expression averaged from 14- to 20-fold higher in AMD vs. normal tissue (Figure 1A–B). Macular *IL17RC* averaged from 47- to 93-fold higher in AMD vs. normal tissue (Figure 1C). Twenty-seven AMD and four normal retinas were assayed in the periphery, but *IL17A* was below the detectable threshold for the qRT-PCR assay in all specimens. *IL17RC* was detectable in 3 (11.1%) AMD peripheral samples, and among these 3 samples, *IL17RC* averaged 20-fold higher in the macula vs. periphery (Figure 1D). *IL17F*, an *IL17A* homologue that has some functional overlap [13], was undetectable in all samples.

Due to the ∼40% yield obtained from the paraffin-embedded slides, we validated our findings using 10 fresh eyes (5 normal, 5 AMD). These AMD donors had early/intermediate AMD pathologies. The macular tissues were paraformaldehyde fixed, cytoprotected, and snap frozen, and thus in significantly better condition for assaying mRNA expression. All samples yielded measurable results. Respectively, *IL17A* and *IL17RC* expression averaged 8.2- and 6.2-fold higher in AMD vs. normal (Figure 1E–F).

Enhanced diffuse immunoreactivity of both IL17A and IL17RC was observed in GA and nAMD (Figure 1G). IL17A immunoreactivity was slightly greater in the macula as compared to the periphery (Figure 1H), but retinal IL17RC was evenly distributed. These results indicate significant aberrant expression of *IL17A* and *IL17RC* mRNA within AMD lesions.

### IL17A is Cytotoxic to ARPE-19

**Figure 2:**
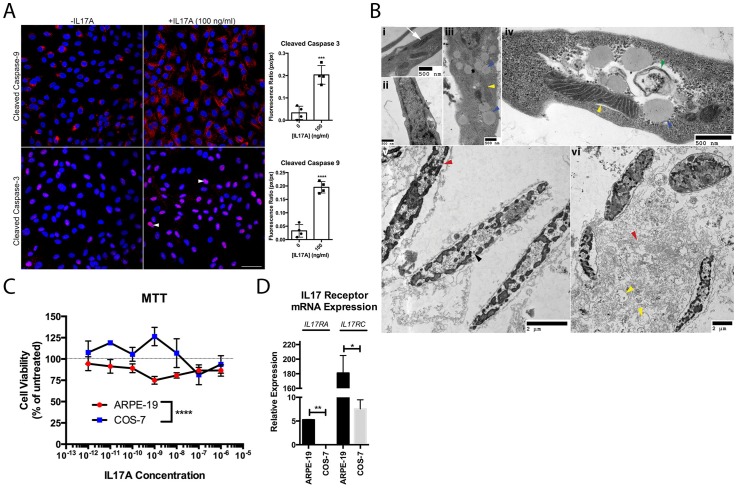
IL17A is cytotoxic to ARPE-19. (A) Confocal microscopy of ARPE-19 treated 48h with IL17A. Nuclear signal of cleaved Caspase-3 (arrowheads). Blue = DAPI. Scale bar = 50 µm. Graphical representation of confocal data presented to the right of the panel. (B) ARPE-19 ultrastructure cultured without (i–ii) and with (iii–vi) 10 ng/ml IL17A for 48h. Indicated are healthy mitochondria (i, white arrow), lipid deposits (iii, iv, blue arrowheads), autophagosomes (iv, green arrowhead), mitochondrial damage (iii, iv, vi, yellow arrowheads), pyknotic nuclear clumps (v, black arrowhead), and cytoplasmic necrosis (vi, red arrowhead). (C) ARPE-19 vs. COS-7 viability after 48h treatment with IL17A. (D) Relative expression of IL17 receptors in ARPE-19 vs. COS-7. Data are presented as mean ± SEM. *: P<0.05, **: P<0.01, ***: P<0.001; ****: P<0.0001.

Degenerative changes to RPE were evaluated in the ARPE-19 cell line. IL17A challenge activated pro-apoptotic Caspase-9 and Caspase-3 (Figure 2A). The pathology of ARPE-19 encompassed lipid accumulations, autophagosomes, mitochondrial damage, nuclear pyknosis, and necrosis (Figure 2B). Viability was assessed by the enzymatic redox of MTT, a mitochondrial reaction requiring NADPH-dependent oxidoreductases, to distinguish cell-type effects of IL17A. IL17A reduced ARPE-19 viability by 25% at 1 ng/ml, whereas COS-7 were relatively unaffected until 100 times that amount was added (Figure 2C). ARPE-19 cells expressed significantly higher levels of IL17RA and IL17RC (Figure 2D), suggesting that ARPE-19 may be more sensitive to IL17A than are COS-7 due to differences in receptor expression.

### IL17RC siRNA Rescues ARPE-19 from IL17A Challenge

**Figure 3:**
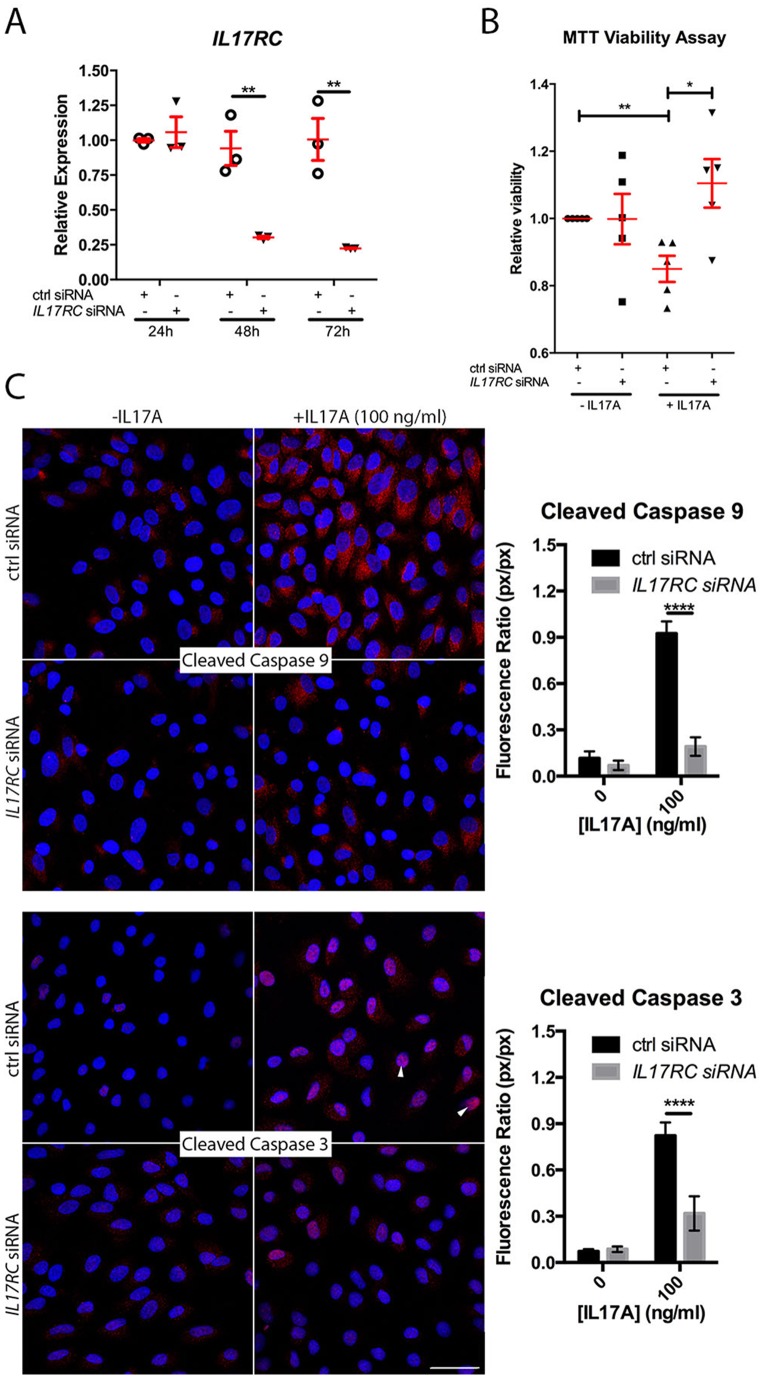
*IL17RC* siRNA prevents IL17A-induced pathology. (A) *IL17RC* knockdown by 0.04 µM siRNA. *IL17RC* was measured by qPCR at 24h, 48h, and 72h post-transfection relative to a universal mRNA standard. (B) Relative viability of ctrl or *IL17RC* siRNA-transfected ARPE-19 with 100 ng/ml IL17A. (C) Confocal microscopy of ctrl or *IL17RC* siRNA-transfected ARPE-19 treated with or without IL17A. Nuclear localization of Caspase-3 (arrowheads). Graphical representation of the Caspase:DAPI ratio are indicated to the right of the images. Blue = DAPI. Scale bar = 50 µm. *: P<0.05; **: P<0.005; ****: P<0.0001. Data are presented as mean ± SEM.

In order to determine whether receptor expression could modulate IL17A cytotoxicity, we knocked down *IL17RC* with siRNA. ARPE-19 were transfected with control (ctrl) or *IL17RC* siRNA prior to IL17A challenge. *IL17RC* siRNA, but not ctrl siRNA, reduced *IL17RC* expression (Figure 3A). *IL17RC* siRNA, but not ctrl siRNA, prevented IL17A-mediated reductions in viability (Figure 3B) and markedly reduced activation of Caspase-9 and Caspase-3 (Figure 3C). We concluded that downregulating receptor expression could prevent IL17-mediated cytotoxicity.

### sIL17R Ameliorates Retinal Degeneration in DKO/rd8 Mice via MAPK

**Figure 4:**
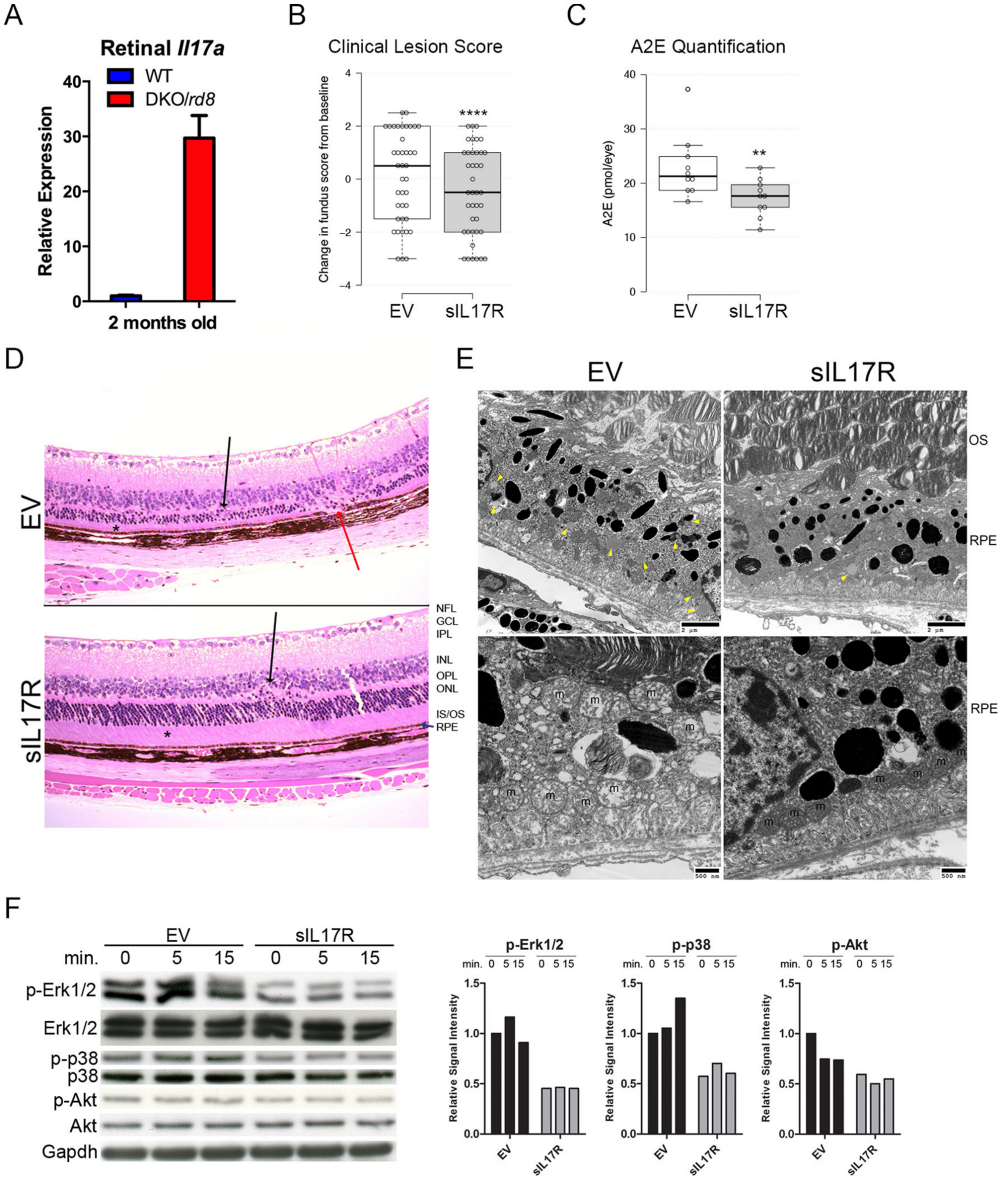
sIL17R prevents retinotoxicity in DKO/*rd8*. (A) Retinal *Il17a* transcripts as a function of animal age. (B) Fundoscopic results 2 months post-intervention. (C) A2E quantification. For box plots: center lines show the medians; box limits indicate the 25th and 75th percentiles as determined by R software; whiskers extend 1.5 times the interquartile range from the 25th and 75th percentiles, outliers are represented by dots; width of the boxes is proportional to the square root of the sample size; data points are plotted as open circles. (D) Histopathological findings: sIL17R preserved photoreceptor IS/OS thickness compared to EV (asterisks) and maintained thickness of the ONL. EV retinas showed RPE degeneration (red arrow). Dystrophic *rd8* lesions are indicated (black arrows). (E) Abundant lipofuscin in EV but not in sIL17R RPE (yellow arrowheads, upper left and right). Mitochondria (m) are damaged and disorganized within EV RPE (lower left) but are viable and organized in sIL17R RPE (lower right). EV RPE show extensive vacuolization, undigested OS and poor basal infoldings (lower left). (F) Western blot of downstream Il17 signaling. EV and sIL17R neuroretinas were treated with 50 ng/ml Il17a for 0, 5, or 15 min *ex vivo*. Relative Signal Intensities are presented of phosphorylated proteins relative to total protein to the right of the blots. NFL = nerve fiber layer; GCL = ganglion cell layer; IPL = inner plexiform layer; INL = inner nuclear layer; OPL = outer plexiform layer; ONL = outer nuclear layer; IS/OS = inner/outer segment; RPE = retinal pigment epithelium *: P<0.05; **: P<0.005; ****: P<0.00001.

We hypothesized that IL17A neutralization could prevent retinal degeneration in DKO/*rd8*. *Il17a* expression in the retina is approximately 30-fold greater in DKO/*rd8* compared to WT at 2 months of age (Figure 4A). To inhibit IL17 in the retina, we administered intravitreal AAV2 vectors encoding soluble IL17 receptor (sIL17R, Figure S1A–B) into right eyes and empty vector (EV) into left eyes of 40 DKO/*rd8* mice over two independent trials and followed them for 2 months. AAV2 vectors preferentially transfect retinal ganglion cells and secreted proteins are detected throughout the entirety of the retina [14], [15]. Injection of AAV2.sIL17R resulted in sIL17R protein expression, no change in retinal *Il17a* transcripts, and a near-significant reduction in *Il6* (Figure S1C–E), supporting sIL17R expression in AAV2.sIL17R- but not in AAV2.EV-treated eyes.

Intervention with sIL17R prevented retinopathy: (i) lesions were fewer by fundoscopy (Figure 4B, Figure S2A–B); (ii) A2E concentration–a lipofuscin fluorophore generated from the visual cycle flux of all-trans-retinal and a biomarker of RPE damage [16]–was reduced (Figure 4C); (iii) photoreceptor atrophy was prevented (Figure 4D, Table S1); (iv) RPE were preserved, evidenced by the lack of vacuolation, lipofuscin, glycogen deposits, undigested photoreceptor outer segments, and mitochondrial atrophy compared to RPE in EV-treated eyes (Figure 4E). The data supports our previous finding that correlated successful anti-inflammatory therapy with reduced *Il17a* expression in DKO/*rd8* retina [9].

We wanted to confirm that sIL17R dampened IL17 signal transduction. IL17A binds to hetero- or homodimers of IL17RA and IL17RC, inducing transcription of pro-inflammatory cytokines via Akt, MAPKs (ERK1/2 and p38), and NF-κB [17]–[19]. We analyzed Erk1/2, p38, and Akt phosphorylation *ex vivo* after challenge with recombinant murine Il17a. Protein lysates were extracted from 12 DKO/*rd8* retinas, 6 expressing EV and 6 expressing sIL17R. Neurosensory retinas were co-incubated with Il17a for 0, 5, or 15 minutes (Figure 4F). There was a 50% reduction in background MAPK and Akt phosphorylation in sIL17R versus EV tissue. Il17a induced p38 and Erk1/2 phosphorylation but was blocked by sIL17R intervention. Akt was not induced in the EV retina, suggesting that Akt had no direct input from Il17a in this tissue. We evaluated NF-κB induction in EV versus sIL17R retinas yet observed no difference in cellular localization (Figure S3). These data corroborate the efficacy of sIL17R and suggest a MAPK-dependent mechanism in the IL17-laden retina.

## Discussion

IL17 was aberrantly expressed in AMD macular lesions compared to non-diseased maculas from controls. This was also true when comparing macular with peripheral expression within AMD retinas. Using a combination of cell and animal studies, we found that IL17 signaling is damaging to cultured RPE and to the retina in a mouse model.

IL17A challenge of ARPE-19 cells reduced cell viability, activated caspase effector proteins, and induced ultrastructural pathology commonly seen in RPE of AMD eyes. Curiously, there was no clear dose-dependent relationship between IL17A concentration and cell viability. Over the course of 5–7 independent experiments using 4–5 replicates per treatment, IL17A nonetheless consistently reduced the viability of ARPE-19 compared to untreated cells. In the case of COS-7, similar reductions were only observed once the cells were co-incubated with a dose 100 times more concentrated than ARPE-19. Using two-way ANOVA to test the validity of these results based upon the two independent variables (here: cell type and IL17A dose), it was significantly different with regard to the viability effects due to changing cell type, but not IL17A dosage. This indicated that ARPE-19 cells and COS-7 cells do indeed respond differently when co-incubated with the cytokine. The effects on viability are nonetheless resolved when examining by TEM. It as this level of detail that various pathological ultrastructures are consistent with changes observed in RPE of AMD lesions: lipid deposits highly reminiscent of the lipofuscin accretions commonly observed in aged RPE and which may potentiate AMD pathophysiology [20]; autophagosomes which are observed in AMD [21]; mitochondrial damage; and nuclear pyknosis and necrosis. Furthermore, siRNA knockdown of IL17RC was sufficient to rescue ARPE-19 viability in the presence of IL17A associated with significant reductions in caspase activation.

The ultimate goal of the present study was to evaluate the therapeutic potential of IL17A knockdown in a preclinical retinal degeneration model. Our lab has generated a mouse model of focal retinal degeneration that undergoes progressive retinal degeneration beginning as early as 2–3 weeks after birth. Despite controversy in recent years [22], the retinal pathology of DKO/*rd8* can be described by two distinct lesions: 1.) “AMD-like” degeneration of RPE and subsequently inner and outer segments of photoreceptors and 2.) “dystrophic/dysplastic” *rd8*-associated lesions affecting inner and outer nuclear layer (INL and ONL) neurons and outer plexiform layer (OPL). AMD-like lesions are associated with dysregulation of the complement system, the RPE, and retinal microglia [23]–[25], all of which are key immunological factors in AMD pathophysiology. Moreover, the elevation of A2E, a biomarker of AMD is measured in DKO/*rd8* eyes [30], [33].

Mice of the C57BL/6N strain (in this study referred to as “WT”), which all possess the *Crb1^rd8^* allele, do not uniformly exhibit signs of retinal dystrophy, as evidenced by wide variations in ophthalmic phenotype within the strain across different research institutions [26]. Furthermore, a *Ccl2^−/−^*/*Cx3cr1^−/−^* strain lacking *Crb1^rd8^* possesses AMD-like lesions caused by age and light-stress [27]. However, DKO/*rd8* is not identical to AMD, especially when noting that anatomically mice lack the macula. Nonetheless, in addition to the above-noted histologic and immunologic features, DKO/*rd8* shares clear IL17 immunopathological features with AMD (Figure 4A).

In DKO/*rd8*, IL17 inhibition using sIL17R, a dominant-negative IL17A inhibitor, was prospectively retinoprotective. This model has a particular allure given the ability to negate all effects due to population variance (even within inbred strains variance is observed [26]) by using the contralateral eye as an internal control [28]. Consequently, between-eye comparisons revealed significant improvements in clinical lesion score, reductions in A2E burden, rescue of retinal architecture histologically and prevention of RPE ultrastructural pathology. Although it is likely that the multiple cell types found within the retina utilize the MAPK signaling cascades towards various ends, it is reassuring to have observed that sIL17R intervention blocked signal transduction via Erk1/2 and p38, a result in line with IL17 signal transduction. Additionally, together with the finding of enhanced ERK1/2 phosphorylation in GA AMD tissue and that Erk1/2 inhibition rescued RPE degeneration in a mouse model [29], these data strengthen the notion that MAPK-mediated IL17 signals are involved in retinal degeneration. Interestingly, sIL17R reduced *Il17rc* retinal transcripts (Figure S2C–D), which, together with the observation that IL17A induces *IL17RA* and *IL17RC in vitro* [30], suggests a positive feedback loop may amplify IL17 signals in susceptible tissues.

Recent efforts have drawn attention to the inflammasome machinery, particularly interleukin-18 (IL-18), as an inciting factor in AMD [31], [32]. Accumulation of toxic *Alu* RNA following loss of DICER1 within RPE activates the NLRP3 inflammasome and consequently IL-18, thus inducing RPE degeneration and contributing to GA pathogenesis [31]. IL-18 and IL-1β, the latter being an inflammasome-induced pro-inflammatory cytokine that is also aberrantly expressed in AMD lesions, have together been shown to induce IL17A [33], [34]. Furthermore, A2E accumulating over time within RPE can serve as a catalyst for IL-1β production via activation of the NLRP3 inflammasome [35]. Consequently, the evolving story of inflammasome activation and IL-18 activity to explain AMD pathology is appealing [36], [37]. However, despite detrimental effects in a mouse model of GA, IL-18 was protective in mouse models of CNV [38]. Given these seemingly contradictory effects, understanding the downstream effectors of the inflammasome machinery is essential towards elucidating possible pathologic drivers and identifying therapeutic targets in AMD [39].

IL17A remains one promising downstream effector of IL-18/IL-1β. Data from our lab show that RPE challenged with NLRP3 secrete IL-18 and IL-1β, and form autophagosomes [40], [41]. Inflammasome-derived IL-18/IL-1β can promote IL17 production from Th17 cells and γδ T cells in a process that is regulated by autophagy [41]. The possibility of ocular resident and/or other cells as the IL17A source in the eyes with AMD requires further investigation given the paucity of observed lymphocytes in AMD lesions [42]. The IL17 pathway is thus poised at the intersection of AMD pathogenesis associated with complement-laden drusen accumulation and inflammasome activation in the macula. The present study indicates that IL17A signaling is both cytotoxic and retinotoxic and should be further considered as a potential therapeutic target in AMD.

## Materials and Methods

### Human Tissue

Tissue investigation was conducted according to Declaration of Helsinki principles and approved by the NIH IRB. Eyes were obtained from the NIH clinical center and Johns Hopkins Wilmer Eye Institute (107 eyes from 67 advanced stage AMD donors and 10 eyes from normal donors), Minnesota Eye Bank (9 normal and 17 AMD), and University of Iowa (5 normal and 5 AMD). Donors signed consent forms from each institution and protocols were approved by each institution’s IRB, as appropriate.

### Cell Culture

ARPE-19 and COS-7 cells (ATCC, Manassas, VA, USA) were used. ARPE-19 were cultured at 37°C with 5% CO_2_ in 10% Fetal Bovine Serum (FBS) DMEM/F12 Ham’s and COS-7 were cultured in 10% FBS/DMEM. Cells were serum-starved without antibiotics for 24 h and treated for 48 h with recombinant IL17A (R&D Systems, Minneapolis, MN).

### Animals

*Ccl2* ^−/−^/*Cx3cr1* ^−/−^/*Crb1^rd8^* mice were generated from the C57BL/6N (*Crb1^rd8^*) background. Where necessary, C57BL/6N are referred to as “WT.” At 4–5 weeks of age, 1.0×10^9^ DNase-Resistant Particles (DRPs) of AAV2.sIL17R were injected into right eyes and 1.0×10^9^ DRPs of AAV2.EV were injected into left eyes of 40 mice in 2 independent trials and followed for 8 weeks. Six mice received the same injections and were used for Western blots. We complied with the Association for Research in Vision and Ophthalmology statement for the use of animals and the NEI’s Institutional Animal Care and Use Committee approved protocols.

### Quantitative Reverse-transcriptase PCR (qRT-PCR)

Human tissues were paraffin-embedded or fresh frozen and RNA was extracted using the Paradise RNA isolation kit (Applied Biosystems, Foster City, CA). For cell and animal studies, total RNA was extracted using Trizol (Qiagen, Hilden, Germany) and chloroform/isopentanol. cDNA was made with the SuperScript II kit (Invitrogen). SYBR Green (Qiagen) primers were used for human *IL17A*, human *IL17RC*, mouse *Il17a*, and mouse *Il17rc.* The TaqMan Gene expression assay (Applied Biosystems) was used for human *IL8* and mouse *Il6*. All data were normalized to the *beta-actin* mRNA level. Expression fold-change was calculated by 2^−ΔΔCT^.

### Immunohistochemistry

Paraffin-embedded human eye sections were deparaffinized, rehydrated, and fixed in acetone. Primary antibodies: IL17A (polyclonal rabbit anti-human IgG, 1∶100 dilution, Catalog #13082-1-AP, Proteintech, Chicago, IL) IL17RC (polyclonal rabbit anti-human IgG, 1∶100 dilution, Catalog #sc-99937, Santa Cruz Biotechnology, Santa Cruz, CA). Secondary antibody: biotin conjugated, goat anti-rabbit IgG (Vector Laboratories, Burlingame, CA). Chromagen: 3,3′-diaminobenzidine (Biocare Medical, Concord, CA). Slides were counterstained with 1% methyl green. For westerns, primary antibodies include phospho- and total-Erk1/2, phospho- and total-Akt, phospho- and total-p38, and GAPDH (Cell Signaling Technology).

### MTT Assay

ARPE-19 or COS-7 cells were plated in quadruplicate on 96-well plates (2×10^4^ cells/well). Cells were serum-starved for 24h then treated for 48h with IL17A. Cells were incubated 4h in 0.5 mg/ml MTT dissolved in DMEM/F12. DMSO was added. Absorption was measured at 570 nm (Synergy II plate reader; Gen5 software; BioTek, Winooski, VT) and normalized to that of untreated cells.

### siRNA Transfection

*IL17RC* small interfering or nonspecific control siRNA RNA (Santa Cruz Biotechnology, Santa Cruz, CA, USA) were transfected at a concentration of 0.04 µM into ARPE-19 cells using transfection reagent according to the manufacturer’s protocol. Transfected cells were serum-starved in media without antibiotics for 24h and treated for 48h with 100 ng/ml IL17A before RNA extraction, immunofluorescence, or MTT assay.

### Soluble Vector Construction and Characterization

Soluble IL17R/9gly/CH3 was cloned into plasmid pCBA(2) [43], which contains hybrid chicken beta-actin (CBA) promoter and bovine growth hormone polyadenylation signal sequence (BGH poly A). The expression cassette was transferred to pre-viral plasmid vector pAAVSP70 containing AAV2 inverted terminal repeats [44]. The recombinant vector AAV2.sIL17R was produced by triple transfection of 293 cells with pAAVSP70.sIL17R and helper plasmids p5repΔCMVcap [45] and pHelper (Stratagene, La Jolla, CA, USA). In empty vector (AAV2.EV), a 3.8 kb intron from alpha-1-antitrypsin replaced sIL17R. Vectors were purified from cell lysates with an iodixanol step gradient and HiTrap Heparin column (GE Healthcare Life Sciences, Piscataway, NJ, USA) on an ÅKTA FPLC system (GE Healthcare Life Sciences, Piscataway, NJ) [45], [46]. Viral vector titers were determined using a TaqMan RT-PCR assay (ABI Prism 7700; Applied Biosystems, Foster City, CA, USA) with primers specific for BGH poly A.

For sIL17R binding to IL17A, ELISA plates were coated with 100 µg/ml human IL17A or mouse IL17A overnight, then blocked with 1% BSA. Serial two-fold dilutions of sIL17R were added in triplicate to the plate and incubated for 1 h at 37°C. Unbound receptor was washed from the plate in an ELISA plate washer. 100 µl biotinylated anti-IL17RA (1 µg/ml) was added to each well and incubated 1 h at room temperature and excess antibody was washed away. Each well was incubated with streptavidin-HRP conjugate and washed. Bound HRP was measured by incubation with TMBD substrate for 20 minutes followed by addition of acid stop solution. OD were measured at 450 nm.

### sIL17R ELISA

Eyes were removed from euthanized mice and stored at −70°C until the assay. The vitreous humor and the retinas were dissected from frozen eyes and homogenized in 200 µL of lysis buffer provided in the ELISA kit. Undiluted homogenates were clarified by centrifugation and assayed for IL17R levels using the human IL17R Duoset kit (R&D Systems).

### Animal Fundoscopy

Fundoscopy was performed before injection and 2 months post-injection. An endoscope with parallel illumination and observation channels was connected to a Nikon D90 digital camera. Mice received intraperitoneal injection of ketamine (1.4 mg/mouse) and xylazine (0.12 mg/mouse) for systemic anesthesia and topical 1% tropicamide ophthalmic solution (Alcon Inc, Fort Worth, TX) for pupil dilation. Grading of the fundoscopic lesions (Table 1) was conducted by a masked observer.

**Table 1:** Clinical scores of the disease.

Progression	+1	>10% increase in retinal lesion number
	+2	>50% increase in size of ≥1/3 of lesions
	+3	>5 fused lesions or appearance of >2 chorioretinal scars
	+4	diffuse chorioretinal scars
Regression	−1	>10% decrease in retinal lesion number
	−2	>50% decrease in size of ≥1/3 of lesions
	−3	>50% disappearance of retinal lesions
	−4	total disappearance of retinal lesions

### A2E Extraction and Quantification

A2E ([2,6-dimethyl-8-(2,6,6-trimethyl-1-cyclohexen-1-yl)-1E,3E,5E,7E-octatetra-enyl]-1-(2-hydroxyethyl)-4-[4-methyl-6(2,6,6-trimethyl-1-cyclohexen-1-yl) 1E,3E,5E,7E-hexatrienyl]-pyridinium) was extracted with chloroform/methanol as previously described [47]. A2E detection and quantification was performed by liquid-chromatography mass spectrometry using a QTRAP 2000 linear ion trap tandem mass spectrometer (Applied Biosystems/MDS SCIEX, Concord, Ontario, Canada) with an Agilent 1100 LC system (Agilent, Wilmington, DE). A gradient of 80% to 98% methanol was used to separate A2E on a C18 column (Zorbax; Agilent) at a flow-rate of 0.3 ml/min. A2E was quantified using external A2E standards.

### Histopathology

Eyes were fixed for 30 min in 4% (v/v) gluteraldehyde followed by 10% (v/v) formalin for 1 h. Fixed eyes were embedded in methacrylate and serially sectioned in the vertical pupillary optic nerve plane. Each eye was cut into four sections and stained with hematoxylin and eosin, then analyzed under a light microscope. For transmission electron microscopy, mouse eyes were fixed in 2.5% gluteraldehyde and 0.5% osmium tetroxide, dehydrated, and embedded into Spurr's epoxy resin. 90 nm sections were made and double-stained with uranyl acetate and lead citrate, and viewed using a JEOL JEM 1010 transmission electron microscope.

### Western Blot

Neurosensory retinas were isolated from 6 DKO/*rd8* mice two weeks after AAV2.sIL17R and AAV2.EV injection. Retinas were cultured 2 h *ex vivo* at 37°C and 5% CO_2_ in 1% BSA in PBS. Retinas were treated with 50 ng/ml recombinant mouse Il17a (R&D Systems) for 0, 5, or 15 min. Protein lysates were isolated in RIPA and complete protein lysis buffer, homogenized using a P200 pipette, and kept on ice 30 min with occasional vortexing. Following centrifugation (16,000 rpm, 30 min, 4°C), we measured protein concentration using the BCA assay (Pierce, Rockford, IL). 10% polyacrylamide gels (Invitrogen) were loaded with 10 µg protein/lane and ran at 125 V for 1 h. Transfer was performed onto a nitrocellulose membrane (Invitrogen) at 300 mA for 1 h at 4°C. Membranes were blocked in 5% BSA for 1 h and incubated with primary antibody overnight at 4°C on a shaker. Membranes were incubated with the secondary antibody goat anti-rabbit conjugated to HRP (1∶10,000) for 45 min at room temperature. Membranes were exposed using SuperSignal West Dura (Pierce).

### Confocal Microscopy

Cells or frozen mouse eye sections were fixed in 4% paraformaldehyde for 15 min, washed in PBS, and blocked in ICC buffer with 5% goat or rabbit serum for 30 min at 4°C. Primary antibody incubation occurred overnight at 4°C. Secondary antibody incubation lasted 1 h at room temperature. Slides were mounted in Vectashield fluorescent media (Vector Labs) and imaged with an Olympus FV1000 Confocal Scanning Scope. Primary antibodies: Cleaved Caspase-3 diluted 1∶200 (Cell Signaling Technology, Danvers, MA), Cleaved Caspase-9 diluted 1∶100 (Santa Cruz), and NF-κB p65 diluted 1∶50 (Cell Signaling Technology). Secondary antibodies: goat anti-rabbit IgG (1∶400), rabbit anti-goat IgG (1∶400), and rabbit anti-mouse IgG (1∶400). DAPI (1∶1000) marked nuclei.

### Statistics

GraphPad was used for statistical tests. Cell and patient qRT-PCR data was evaluated using the unpaired T-test. MTT assays were compared using two-way ANOVA. For mean fluorescence quantification, the ratios of secondary antibody (red signal) to DAPI (blue signal) were compared by two-way ANOVA. For mouse qRT-PCR, clinical fundus scores, and A2E, the paired T-test was used. *P*<0.05 determined statistical significance.

## Supporting Information

Figure S1 **AAV2.sIL17R vector design and efficacy.** (A) AAV2.sIL17R domains. (B) sIL17R binding data for mouse and human cytokine presented. (C) ELISA quantification of sIL17R protein in mouse retina 2 months post-injection. qRT-PCR measurement of retinal (D) *Il17a* and (E) *Il6* mRNA. *: P<0.05; n.s. = not significant.
(JPEG)

Figure S2 **sIL17R vector attenuates retinal lesions by clinical and molecular evaluation.** (A) Representative fundus images comparing baseline to 2 months post-injection of EV or sIL17R vector with lesion score indicated to the right. (B) Intra-mouse pairwise comparison of lesion scores, EV vs. sIL17R (80 eyes total from 40 mice); all points to the right of the red line indicate that the sIL17R-treated eye faired better than its contralateral counterpart. Lower *Il17rc* transcript expression in sIL17R vs. EV retinas 2 months post-injection by qRT-PCR (C) and end-stage gel (D).
(JPEG)

Figure S3 **Cellular Localization of NF-κB in DKO/** ***rd8*** **.** Confocal microscopy of immunolabeled frozen eye sections from EV and sIL17R eyes 2 months post-intervention. INL = inner nuclear layer, ONL = outer nuclear layer.
(JPEG)

Table S1 **Histopathologic results.** Clinical and histologic descriptions of all mice used in the study.
(JPEG)
