# Supplementary figures and images for: Interleukin-17 Retinotoxicity Is Prevented by Gene Transfer of a Soluble Interleukin-17 Receptor Acting as a Cytokine Blocker: Implications for Age-Related Macular Degeneration
Source: PLoS One. 2014 Apr 29;9(4):e95900. doi: 10.1371/journal.pone.0095900 (PMC4004582; doi:10.1371/journal.pone.0095900)

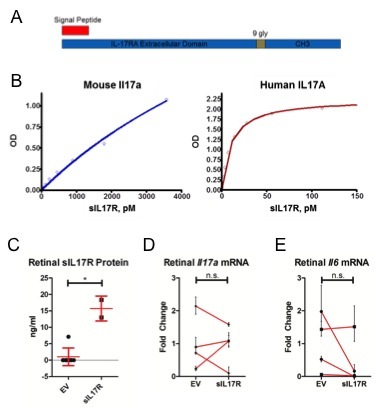

Supplement: Figure S1 — AAV2.sIL17R vector design and efficacy. (A) AAV2.sIL17R domains. (B) sIL17R binding data for mouse and human cytokine presented. (C) ELISA quantification of sIL17R protein in mouse retina 2 months post-injection. qRT-PCR measurement of retinal (D) Il17a and (E) Il6 mRNA. *: P<0.05; n.s. = not significant. (JPEG) [file pone.0095900.s001.jpeg]

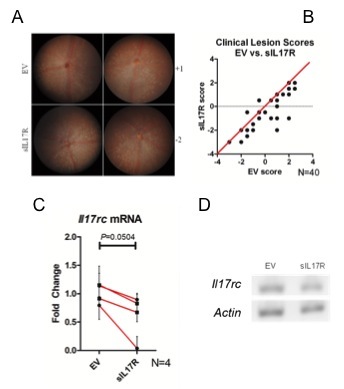

Supplement: Figure S2 — sIL17R vector attenuates retinal lesions by clinical and molecular evaluation. (A) Representative fundus images comparing baseline to 2 months post-injection of EV or sIL17R vector with lesion score indicated to the right. (B) Intra-mouse pairwise comparison of lesion scores, EV vs. sIL17R (80 eyes total from 40 mice); all points to the right of the red line indicate that the sIL17R-treated eye faired better than its contralateral counterpart. Lower Il17rc transcript expression in sIL17R vs. EV retinas 2 months post-injection by qRT-PCR (C) and end-stage gel (D). (JPEG) [file pone.0095900.s002.jpeg]

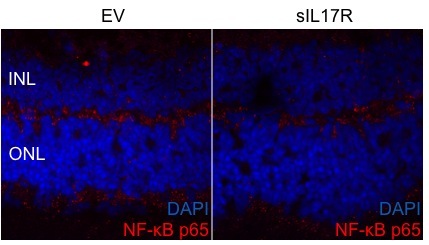

Supplement: Figure S3 — Cellular Localization of NF-κB in DKO/ rd8 . Confocal microscopy of immunolabeled frozen eye sections from EV and sIL17R eyes 2 months post-intervention. INL = inner nuclear layer, ONL = outer nuclear layer. (JPEG) [file pone.0095900.s003.jpeg]

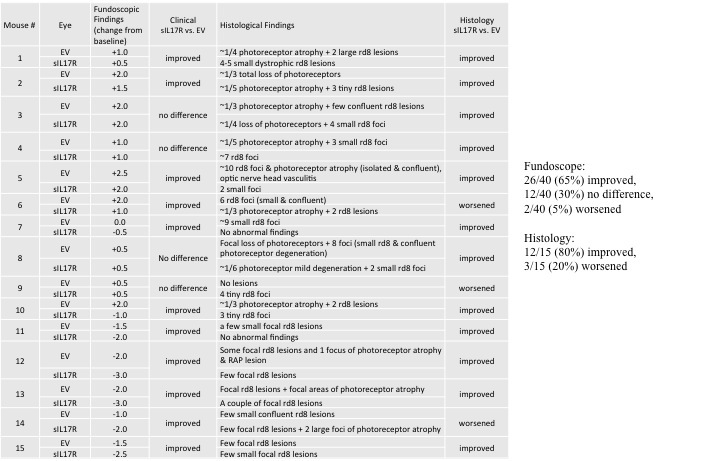

Supplement: Table S1 — Histopathologic results. Clinical and histologic descriptions of all mice used in the study. (JPEG) [file pone.0095900.s004.jpeg]
